# Natural Product-Based Therapeutic Potential of *Fagonia arabica*: Phytochemical Characterization and Biological Activities of Leaf and Seed Extracts

**DOI:** 10.3390/ph19091330

**Published:** 2026-08-23

**Authors:** Ibrahim M. Aziz, Rawan M. Alshalan, Reem M. Aljowaie

**Affiliations:** Department of Botany and Microbiology, College of Science, King Saud University, P.O. Box 2455, Riyadh 11451, Saudi Arabia; ralshalaan@ksu.edu.sa (R.M.A.); raljowaie@ksu.edu.sa (R.M.A.)

**Keywords:** *Fagonia arabica*, phytochemicals, bioactive compounds, biological activities, natural products, therapeutic potential

## Abstract

**Background/Objectives**: The increasing global prevalence of chronic diseases has intensified the search for potential natural therapeutic agents. *Fagonia arabica* is a medicinal plant widely used in traditional medicine; however, comparative information on the phytochemical composition and biological activities of its leaves and seeds remains limited. To the best of our knowledge, this study represents the first comparative investigation of the phytochemical profiles and bioactivities of methanolic leaf (FALE) and seed (FASE) extracts of *F. arabica* collected from Riyadh, Saudi Arabia. **Methods**: Methanolic extracts were analyzed by gas chromatography–mass spectrometry (GC–MS), and their total phenolic content (TPC), total flavonoid content (TFC), antioxidant, antimicrobial, anticancer, and antidiabetic activities were evaluated using standard *in vitro* assays. **Results**: GC–MS analysis identified 60 and 68 compounds in FALE and FASE, respectively, with (*9Z*,*12Z*,*15Z*)-octadeca-9,12,15-trienoic acid, hexadecanoic acid, dimethyl-2,3,4,7,8,9,11,12,14,15,16,17-dodecahydro-*1H*-cyclopenta[a]phenanthren-3-ol, and (*1R*,*3aR*,*5aR*,*5bR*,*7aR*,*9S*,*11aR*,*11bR*,*13aR*,*13bR*)-3a,5a,5b,8,8,11a-Hexamethyl-1-(prop-1-en-2-yl)-1,2,3,4,5,6,7,7a,9,10,11,11b,12,13,13a,13b-hexadecahydrocyclopenta[a]chrysen-9-ol as major constituents. FASE exhibited higher TPC and TFC values (104.13 mg GAE/g and 62.16 mg QE/g, respectively) than FALE. Both extracts demonstrated antioxidant activity, with FASE showing greater potency against DPPH and ABTS radicals (IC_50_ = 63.3 and 42.4 μg/mL, respectively). FASE also displayed broad-spectrum antimicrobial activity, particularly against *Staphylococcus aureus* (MIC = 7.81 μg/mL). Furthermore, FASE exhibited stronger concentration-dependent cytotoxicity against MCF-7 and HepG2 cells, with IC_50_ values of 106.8 and 89.5 μg/mL, respectively, compared with 112.5 and 103.1 μg/mL for FALE. Apoptosis induction in MCF-7 and HepG2 cells was associated with upregulation of caspase-3, caspase-8, caspase-9, and *Bax*, alongside downregulation of *Bcl-2* and *Bcl-xL*. In addition, FASE exhibited potent *α*-amylase and *α*-glucosidase inhibitory activities (IC_50_ = 61.62 and 67.38 μg/mL, respectively), comparable to acarbose. **Conclusions**: These findings highlight *F. arabica* seed extract as a promising source of multifunctional bioactive compounds with potential pharmaceutical application.

## 1. Introduction

Natural products have long been a cornerstone of drug discovery, providing a vast chemical diversity that continues to inspire the development of new therapies [1]. *Fagonia arabica* L. (family *Zygophyllaceae*) is a small, spiny, densely branched, perennial undershrub that can reach a height of 30 to 60 cm [2]. It is a species with extensive use in traditional medicine throughout desert regions such as Saudi Arabia, Pakistan, India, and South Africa [2]. This plant possesses thin, angular stems, pinnately compound leaves with 3–5 pairs of leaflets, small pinkish-purple flowers, and fruit that is a pendulous capsule containing many seeds [2,3]. It has been used ethnobotanically to treat a variety of conditions, including sore mouth and smallpox, as well as inflammatory, hematological, and endocrinological problems [3]. This traditional use is supported by a diverse array of phytochemicals, including glycosides, flavonoids, terpenoids, saponins, and alkaloids [2]. Thus, Iftikhar et al. (2022) conducted a detailed study of the main groups of bioactive compounds in *F. arabica*, including glycosides, flavonoids, terpenoids, saponins, and alkaloids [2]. Another study isolated and identified flavonoids in the aerial parts of *F. arabica*, namely kaempferol-7-O-rhamnoside and acacetin-7-O-rhamnoside [4]. Mahmoud et al. (2025) investigated the phytochemical composition and biological activity of the floral parts of *F. arabica* in Egypt [3]. Other studies have examined other *Fagonia* species, such as *F. cretica* [5], *F. indica* [6], and *F. longispina* [7]. As a result, *F. arabica* has been the subject of numerous pharmacological studies, revealing a variety of actions, including antioxidant, antibacterial, cardioprotective, and anticancer properties. However, much of the existing research has focused on whole-plant or leaf extracts, leaving a considerable gap in our understanding of the relative bioactivity of various plant parts.

While previous studies have highlighted the medicinal potential of *F. arabica*, a detailed comparative investigation of the phytochemical composition and biological activities of methanolic leaf (FALE) and seed (FASE) extracts collected from Riyadh, Saudi Arabia, has not been reported. This study was designed to compare leaf and seed aqueous and methanolic extracts of *F. arabica* with respect to their phytochemicals, antioxidant, antimicrobial, anticancer, and antidiabetic activities. A gas chromatography–mass spectrometry (GC–MS) analysis was performed to identify bioactive compounds, and a series of *in vitro* bioassays was carried out to evaluate the various biological activities. The objectives of this study were to compare the two extracts, identify the most active one, elucidate possible mechanisms of action, and explain why one part of the plant is more active than the other, thereby informing the development of targeted phyto-medicines for the treatment of various diseases. The findings of the study would contribute to the medical field by identifying novel therapeutic agents from a readily available desert tree, *F. arabica*.

## 2. Results

### 2.1. Extraction Yields

The extraction yields of FALE and FASE were 10.14 ± 1.65% and 12.05 ± 2.16% (*w*/*w*), corresponding to 2.03 ± 0.33 g and 2.41 ± 0.43 g of dried crude extract, respectively, from 20 g of dried plant material.

### 2.2. GC–MS Analysis

**Figure 1:**
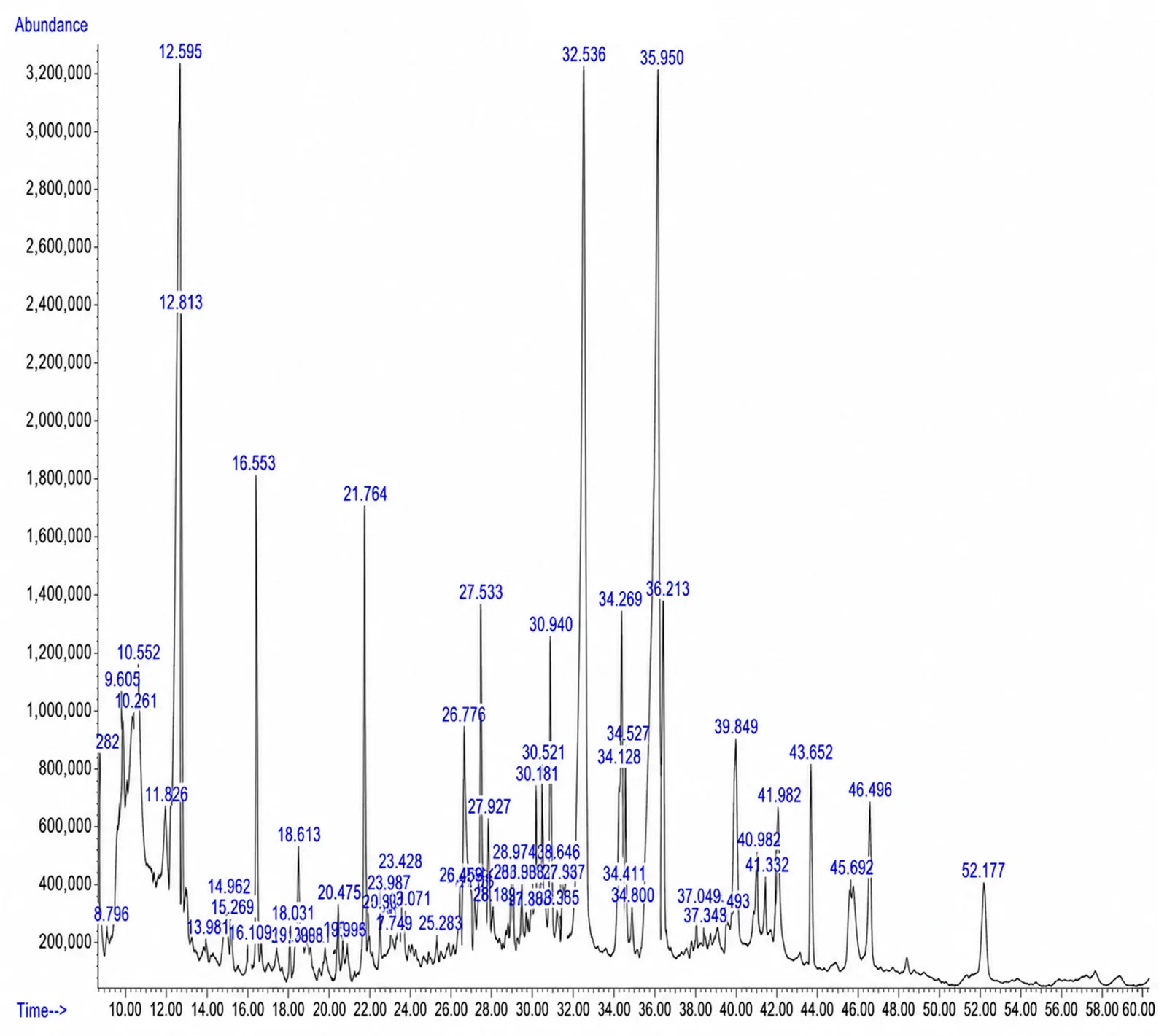
FALE GC–MS chromatogram displaying the distribution of detected chemicals. The prominent peak corresponds to the main molecule in the extract, and each spectral peak represents a distinct phytochemical.

**Figure 2:**
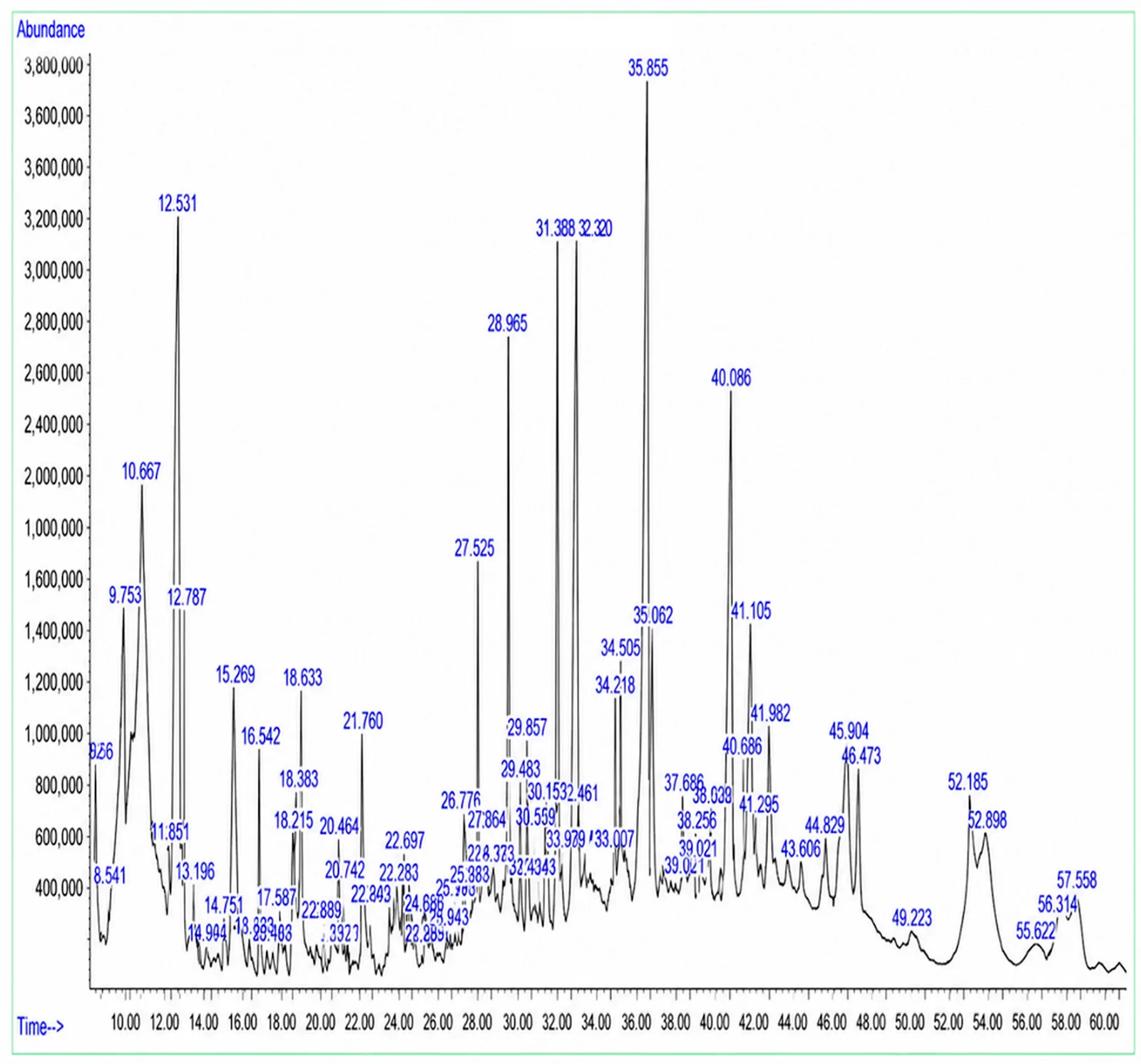
FASE GC–MS chromatogram displaying the distribution of detected chemicals. The prominent peak corresponds to the main molecule in the extract, and each spectral peak represents a distinct phytochemical.

The bioactive compounds of FALE and FASE were identified using GC–MS. The chromatograms are presented in Figure 1 and Figure 2, along with details such as retention time (RT), peak area (%), molecular formula (MF), and molecular weight (MW), are summarized in Table 1 and Table 2. The analysis found many different chemicals in both extracts, with some similarities and differences. FALE had 60 compounds, with the main ones being (*9Z*,*12Z*,*15Z*)-octadeca-9,12,15-trienoic acid (14.22%), 3,5-Dihydroxy-6-methyl-2,3-dihydro-4H-pyran-4-one (12.75%), and hexadecanoic acid (12.57%). Some terpenoids and fatty acids, like (*E*,*7R*,*11R*)-3,7,11,15-tetramethylhexadec-2-en-1-ol (1.33%) and (*2Z*)-2-(3,3-dimethyl-2-bicyclo [2.2.1]heptanylidene)ethanol (1.16%), were also found. These suggest possible antioxidant and anti-inflammatory effects.

FASE had 68 compounds, showing more complexity than FALE. The main ones were 2-methylhexanoic acid (13.43%) and (*9Z*,*12Z*,*15Z*)-octadeca-9,12,15-trienoic acid (10.33%). It also contained important compounds such as dimethyl-2,3,4,7,8,9,11,12,14,15,16,17-dodecahydro-*1H*-cyclopenta[a]phenanthren-3-ol (2.61%) and (*1R*,*3aR*,*5aR*,*5bR*,*7aR*,*9S*,*11aR*,*11bR*,*13aR*,*13bR*)-3a,5a,5b,8,8,11a-Hexamethyl-1-(prop-1-en-2-yl)-1,2,3,4,5,6,7,7a,9,10,11,11b,12,13,13a,13b-hexadecahydrocyclopenta[a]chrysen-9-ol (1.34%), known for their health benefits. Both extracts shared several major compounds, but FASE had more fatty acids and unique compounds such as *β*-amyrin. This suggests FASE might have more health benefits. Overall, both extracts are rich in active chemicals, but FASE has greater variety and more important compounds. This might explain why they have different effects.

The percentage area values presented in Table 1 and Table 2 represent the relative abundance of the identified compounds as a percentage of the total ion chromatogram (TIC) area of all detected compounds. It should be noted that not all components of the extract may be amenable to GC–MS analysis under the conditions employed, and therefore, the reported percentages reflect the composition of the identified compounds only, not the entire extract mixture. However, it is important to note that not all the constituents in the extract can necessarily be analyzed by the use of GC–MS, and as such, the percentages are only indicative of the components that were found through the analysis.

### 2.3. Total Phenolic Content (TPC) and Total Flavonoid Content (TFC)

The TPC and TFC of FALE and FASE were assessed to determine their phytochemical richness and antioxidant potential. Notably, FASE demonstrated higher TPC (104.13 ± 1.25 mg GAE/g DW) and TFC (62.16 ± 1.34 mg QE/g DW) values compared to FALE, which exhibited TPC and TFC values of 95.18 ± 1.67 mg GAE/g DW and 56.19 ± 1.94 mg QE/g DW, respectively. This indicates that FASE is richer in phenolic and flavonoid compounds.

### 2.4. Antioxidant Activity of FALE and FASE

**Figure 3:**
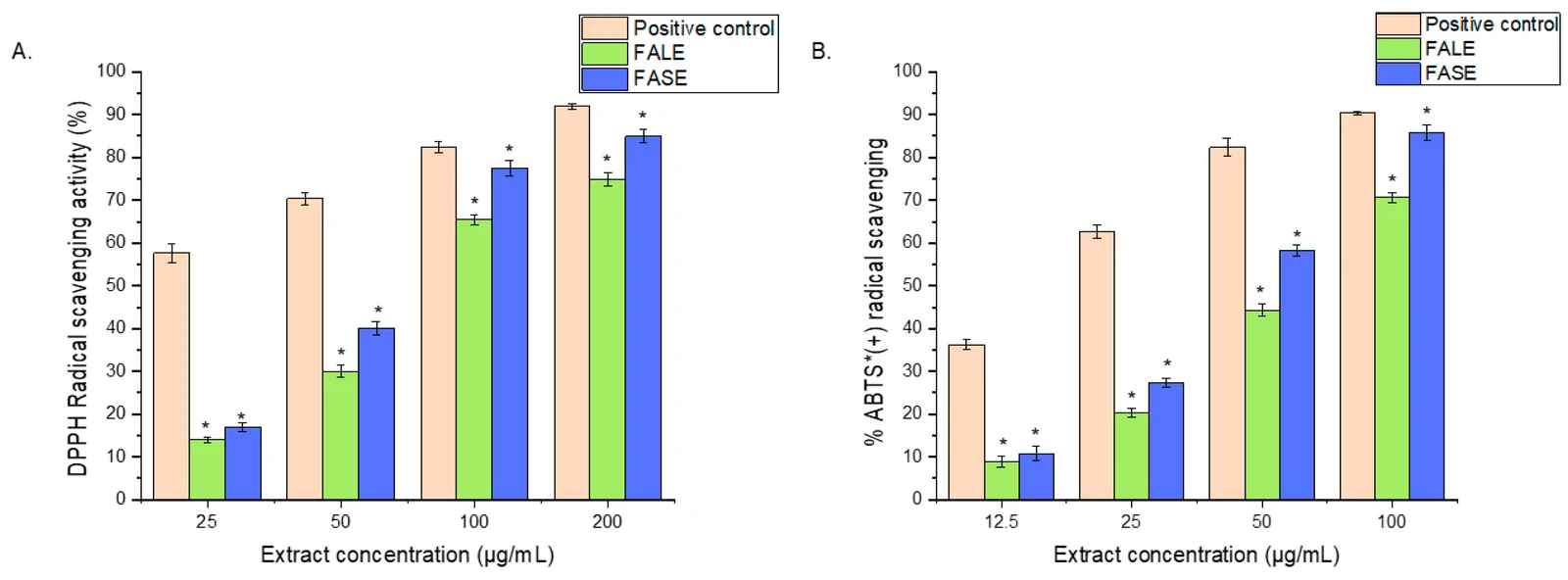
Antioxidant activity of FALE and FASE: (**A**) DPPH and (**B**) ABTS^+^ assays. Ascorbic acid served as the positive control. Data are mean ± SD (*n* = 3). * *p* < 0.05 vs. positive control (ascorbic acid). (+), radical cation.

As shown in Figure 3A,B, the antioxidant properties of FALE and FASE were assessed using 2,2-diphenyl-1-picrylhydrazyl (DPPH) radical scavenging activity and 2,2’-azinobis-(3-ethylbenzothiazoline-6-sulfonic acid) (ABTS*+) radical-scavenging assays, in contrast to the conventional antioxidant positive control (ascorbic acid). Both tests showed a concentration-dependent increase in free radical scavenging activity, suggesting that the extracts contained strong antioxidant components. The scavenging activity in the DPPH assay rose as concentrations rose from 25 to 200 µg/mL. At 200 µg/mL, FALE showed 74.93 ± 1.64% inhibition, and FASE showed 84.93 ± 1.54%, whereas the positive control (ascorbic acid) showed 91.96 ± 0.65%. The higher antioxidant effectiveness of FASE in the DPPH assay was demonstrated by the calculated IC_50_ values of 63.3 µg/mL for FASE and 78.2 µg/mL for FALE. Similarly, both extracts demonstrated significant (*p* < 0.05) radical-scavenging activity in the concentration-dependent ABTS^+^ assay. FASE inhibited 85.88 ± 1.79% at 100 µg/mL, while FALE inhibited 70.64 ± 1.14%, and the positive control inhibited 90.36 ± 0.36%. For FASE and FALE, the IC_50_ values were roughly 42.4 µg/mL and 57.4 µg/mL, respectively. Overall, the findings imply that both extracts have considerable antioxidant activity, especially FASE, likely due to its more complex phytochemical composition.

### 2.5. Antimicrobial Effects of FALE and FASE

The antibacterial and antifungal activities of FALE and FASE against various bacteria were evaluated using disk diffusion assays (Table 3 and Table 4), and against selected *Candida* strains (Table 5 and Table 6). Both extracts demonstrated significant, concentration-dependent inhibitory effects; increasing extract concentrations (125–1000 µg/mL) resulted in lower minimum inhibitory concentration (MIC) and minimum bactericidal concentration (MBC) values (*p* < 0.05) and increased zones of inhibition (ZoI). The extracts showed broad-spectrum antibacterial potential, although their activities were lower than those of conventional chloramphenicol.

Among the analyzed extracts, FASE demonstrated superior antibacterial efficacy compared with FALE. It produced larger ZoI and lower MIC/MBC values against both Gram-positive and Gram-negative bacteria. *Staphylococcus aureus* and *Enterococcus faecalis* were the most susceptible bacterial strains, with MIC values ranging from 7.81 ± 0.36 to 15.63 ± 1.22 µg/mL. Conversely, *Pseudomonas aeruginosa*, *Klebsiella pneumoniae*, and *Escherichia coli* exhibited relatively higher resistance. 

Similarly, both extracts demonstrated significant antifungal activity against *Candida albicans*, *C. glabrata*, and *C. parapsilosis*. FASE exhibited stronger anticandidal activity than FALE, with the highest inhibition observed against *C. parapsilosis* (20 ± 2 mm). Overall, the results highlight the promising antimicrobial and antifungal potential of both extracts, particularly FASE.

### 2.6. Anticancer Activity and Apoptosis-Related Markers

**Figure 4:**
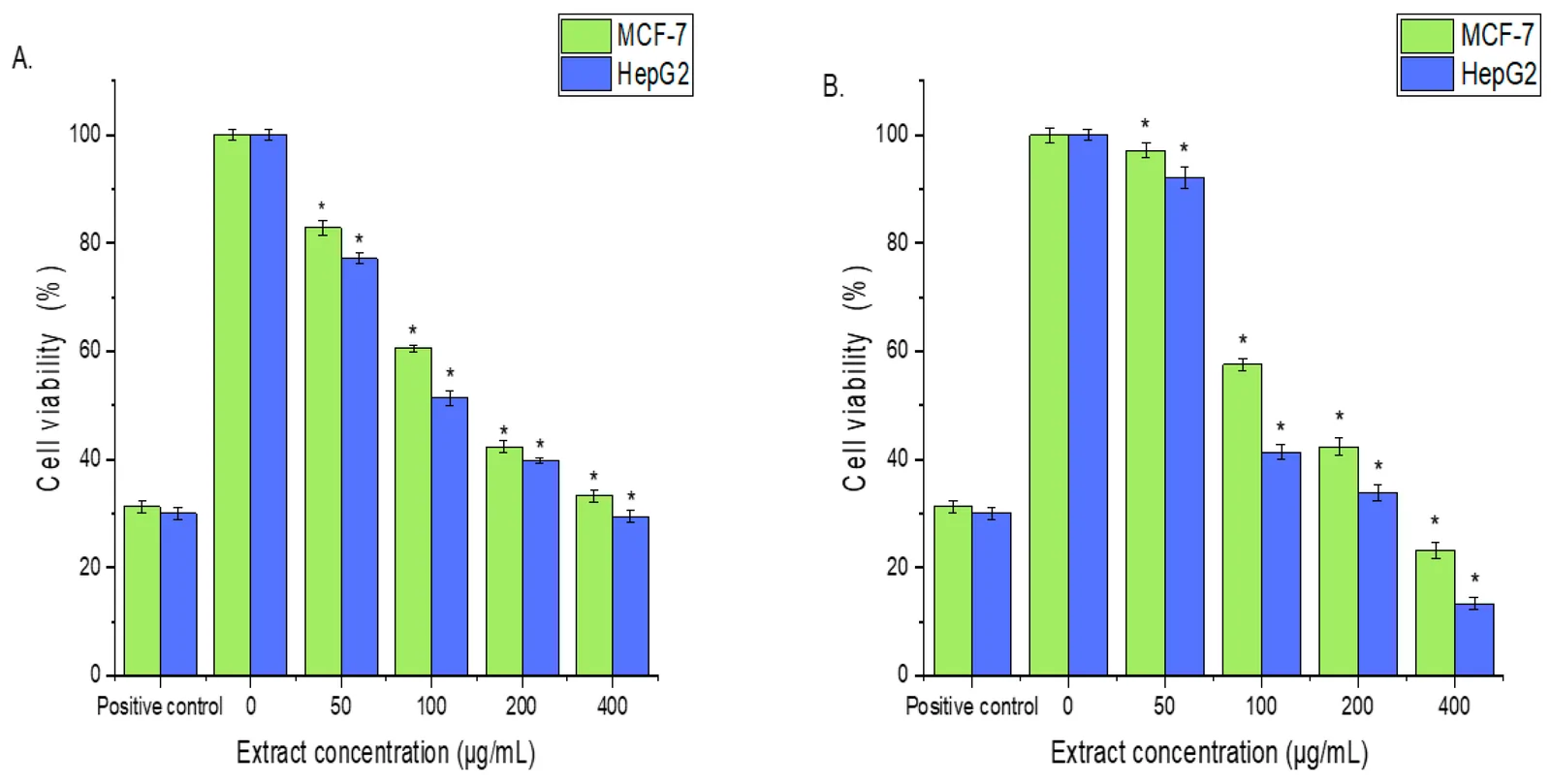
Effects of FALE (**A**) and FASE (**B**) on MCF-7 and HepG2 cell viability assessed by MTT assay following 24 h treatment (0–400 µg/mL). Values are mean ± SD (*n* = 3). * *p* < 0.05 versus positive control (cisplatin (30 µg/mL).

The 3-[4,5-dimethylthiazol-2-yl]-2,5-diphenyltetrazolium bromide (MTT) assay was used to assess the anticancer activities of FALE and FASE against MCF-7 and HepG2 cells (Figure 4A,B). Both extracts showed concentration-dependent cytotoxicity, with cell viability decreasing as extract concentration increased. FASE demonstrated stronger anticancer activity than FALE, particularly against HepG2 cells. The estimated IC_50_ values for FALE were 112.5 μg/mL and 103.1 μg/mL against MCF-7 and HepG2 cells, respectively, whereas FASE exhibited lower IC_50_ values of 106.8 μg/mL and 89.5 μg/mL against MCF-7 and HepG2 cells, respectively. At the highest tested concentration (400 µg/mL), FALE reduced the viability of MCF-7 and HepG2 cells to 33.29 ± 1.16% and 29.43 ± 1.136%, respectively, while FASE reduced cell viability to 23.29 ± 1.45% in MCF-7 cells and 13.42 ± 1.136% in HepG2 cells, indicating its superior cytotoxic potential against cancer cells. 

**Figure 5:**
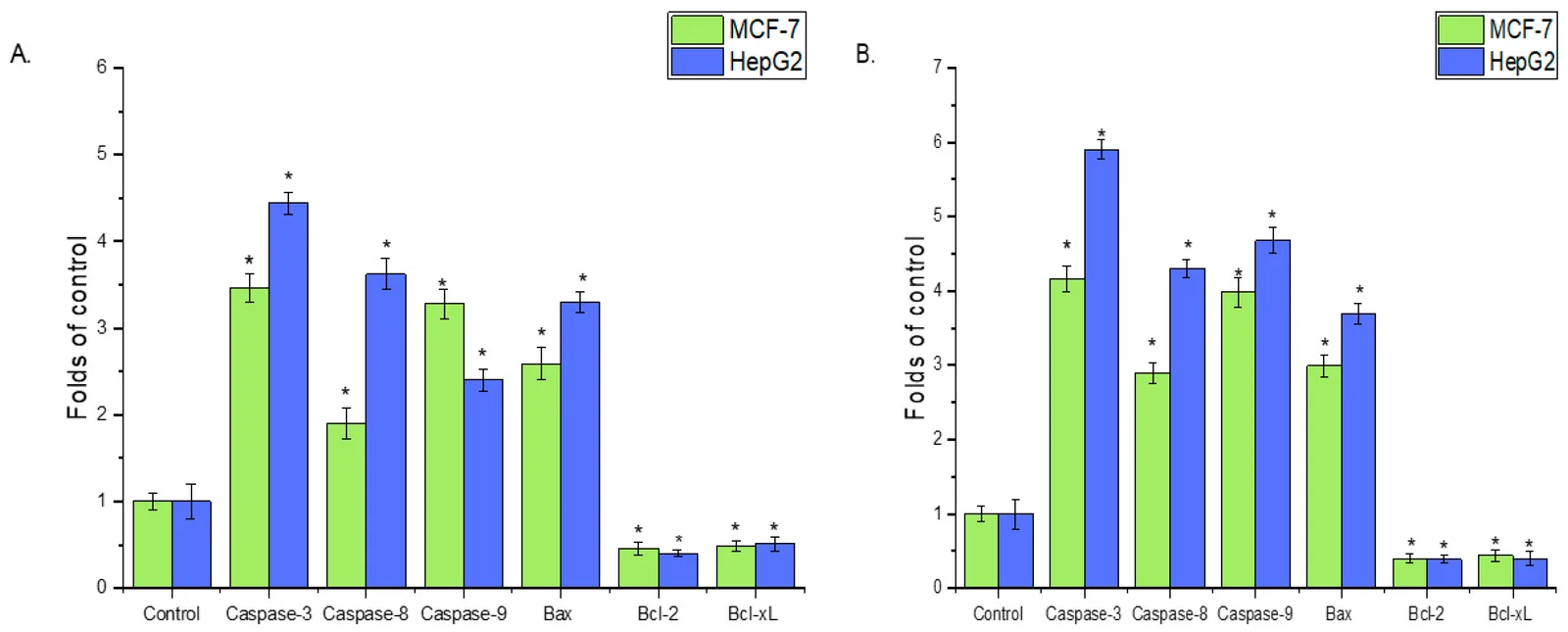
Effects of FALE (**A**) and FASE (**B**) on apoptosis-related gene expression in MCF-7 and HepG2 cells after 48 h treatment at IC_50_ concentrations. FALE and FASE increased *caspase-3*, *caspase-8*, *caspase-9*, and *Bax* expression and decreased *Bcl-2* and *Bcl-xL* expression. Data are presented as mean ± SD (*n* = 3). * *p* < 0.05 vs. untreated control cells.

FALE and FASE exhibited significant anticancer activity against MCF-7 and HepG2 cells through concentration-dependent cytotoxic and pro-apoptotic effects. Quantitative real-time PCR (qRT-PCR) analysis (Figure 5A,B) confirmed that both extracts induced apoptosis by significantly upregulating pro-apoptotic markers, including *caspase-3*, *caspase-8*, *caspase-9*, and *Bax*, while downregulating the anti-apoptotic markers *Bcl-2* and *Bcl-xL* compared with the control group. FASE showed stronger apoptotic activity than FALE, particularly in HepG2 cells, where *caspase-3* and *Bax* expression reached 5.90 ± 0.13-fold and 3.69 ± 0.14-fold, respectively. Overall, the findings indicate that FALE, and especially FASE induce apoptosis through intrinsic and extrinsic pathways, highlighting their potential as natural anticancer agents.

### 2.7. Antidiabetic Activity

**Figure 6:**
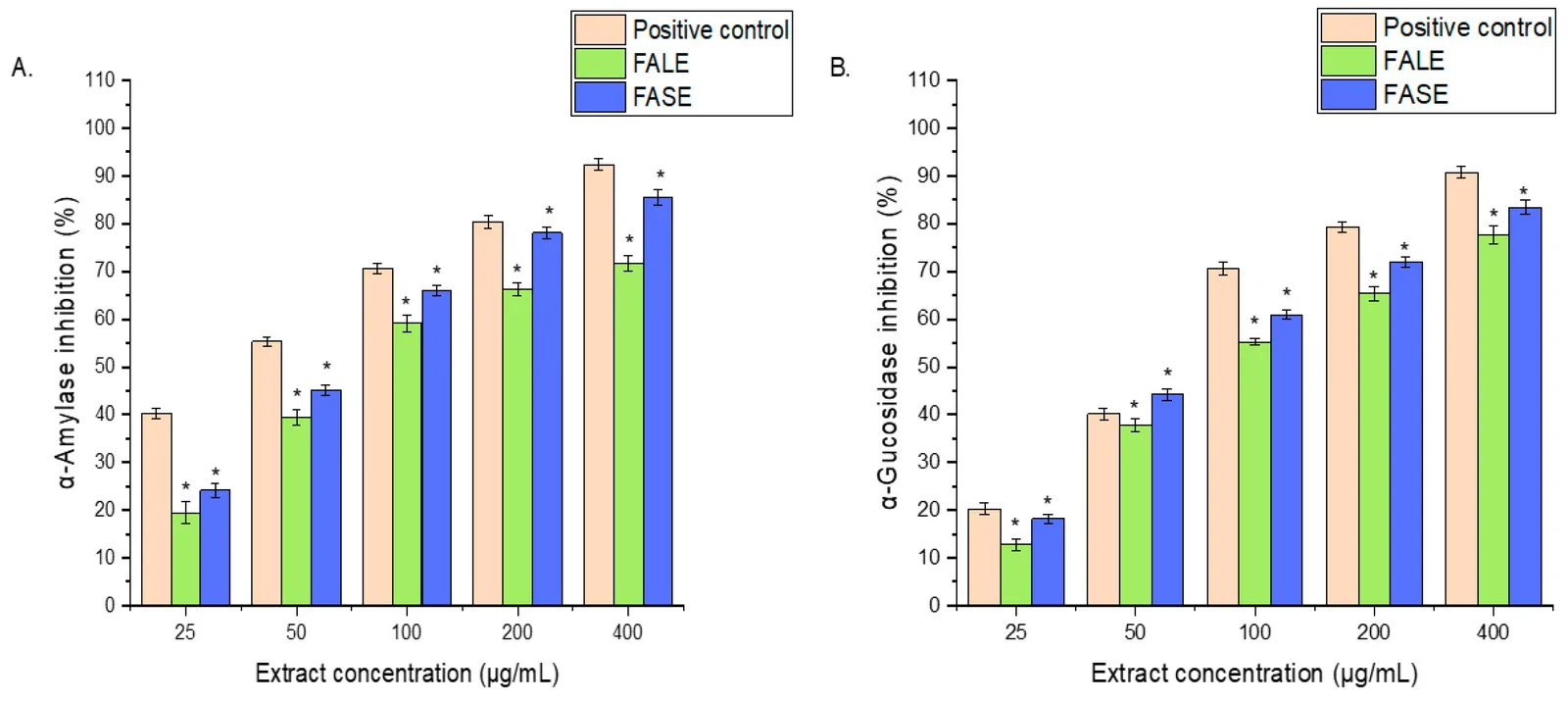
Inhibitory effects of FALE and FASE on (**A**) α-amylase and (**B**) α-glucosidase activities at concentrations ranging from 25 to 400 µg/mL. Values are expressed as mean ± SD (*n* = 3). * *p* < 0.05 versus the positive control (acarbose).

The antidiabetic potential of FALE and FASE was evaluated by assessing their inhibitory activities against α-amylase and α-glucosidase. Both extracts showed a clear concentration-dependent inhibitory effect across the tested concentrations (50–400 µg/mL), as shown in Figure 6A,B. The IC_50_ values indicated that FASE exhibited stronger inhibitory activity than FALE in both assays. In the α-amylase inhibition assay, FASE exhibited an IC_50_ of 61.62 µg/mL, whereas FALE showed an IC_50_ of 76.75 µg/mL. The positive control (acarbose) exhibited the highest inhibitory effect, with an IC_50_ of 41.20 µg/mL. Likewise, in the α-glucosidase assay, FASE demonstrated greater inhibitory potency, with an IC_50_ of 67.38 µg/mL, compared to 85.06 µg/mL for FALE, while acarbose recorded an IC_50_ of 66.13 µg/mL. Overall, the results suggest that both extracts possess promising antidiabetic activity, with FASE exhibiting superior inhibitory effects against carbohydrate-hydrolyzing enzymes compared with FALE.

## 3. Discussion

The percentage yield of the *F. arabica* extracts was favorable. The FALE extraction yielded an average of 10.14 ± 1.65% while the FASE extraction yielded an average of 12.05 ± 2.16 (*w*/*w*). The percent yields obtained from the FASE in this study were in good agreement with previously reported percent yields from *F. arabica* seeds from Saudi Arabia and neighboring countries, as well as from aerial parts from elsewhere in the world using methanolic extraction. The percent yields were found to range from 8–15% (*w*/*w*) and are greatly affected by the type of plant material that is being extracted, as well as the solvents that are used for the extraction process, and finally, the geographical location in which the plant is being grown [2]. In a previous study, the percent yield of an ethanolic extract from the seeds of an ecotype of *F. arabica* from Saudi Arabia was only 8.5% (*w*/*w*) [8]. This clearly demonstrates the superiority of the aqueous methanolic extraction method used in this study for extracting a diverse group of solubilized seed compounds from FASE.

The phytochemical profiles of FASE and FALE were established by GC–MS. FASE contains a complex mixture of 68 compounds, whereas FALE contains 60. FASE is therefore a rich source of a variety of bioactive compounds. Among these, the major compounds in both extracts were identified as (*9Z*,*12Z*,*15Z*)-octadeca-9,12,15-trienoic acid (14.22% in FALE and 10.33% in FASE), hexadecanoic acid, dimethyl-2,3,4,7,8,9,11,12,14,15,16,17-dodecahydro-*1H*-cyclopenta[a]phenanthren-3-ol (2.61% in FASE), (*1R*,*3aR*,*5aR*,*5bR*,*7aR*,*9S*,*11aR*,*11bR*,*13aR*,*13bR*)-3a,5a,5b,8,8,11a-Hexamethyl-1-(prop-1-en-2-yl)-1,2,3,4,5,6,7,7a,9,10,11,11b,12,13,13a,13b-hexadecahydrocyclopenta[a]chrysen-9-ol (1.34% in FASE), and many other flavonoids and terpenoids. The majority of these compounds were previously reported in other species of the genus *Fagonia*. A detailed review of the phytochemicals of *F. arabica* reported the major classes of bioactive compounds, including glycosides, flavonoids, terpenoids, saponins, alkaloids, and trace elements [4]. Some of the specific flavonoids isolated and identified were kaempferol-7-O-rhamnoside and acacetin-7-O-rhamnoside. Recent studies have used high-performance liquid chromatography (HPLC) analysis of aerial parts of *F. arabica*, which revealed the presence of tannins, steroids, diterpenes, saponins, flavonoids, and alkaloids. Malavika et al. (2021) conducted an extensive study of the chemical and pharmacological properties of the genus *Fagonia*, emphasizing the diverse range of biologically active compounds across species [9]. From the *Zygophyllaceae* family, it is well known that seed tissues of the plants of this family contain higher amounts of defense-related secondary metabolites than leaves of the same plant [10]. The GC–MS analysis of *F. longispina* (*Zygophyllaceae*) showed richness in aliphatic ketones such as *cis*-4-(4’-T-butylcyclohexyl)-4-methyl-2-pentanone (33.33%), cyclohexyl-2-methylene butanyl ketone (14.28%), and *trans*-4-(–t-butylcyclohexyl)-4-methyl-2-pentanone (9.52%), and in cyclic terpenoids such as 4*β*-(*tert*-butyl)-1*α*-(1-methylvinyl)cyclohexanemethanol (9.52%), and Isoprenoid such as 2,6,10-trimetyl, 14-ethylne-14 pentadecene (6.66%) [7]. The GC–MS analysis of *Zygophyllum coccineum* essential oil revealed a high concentration of sesquiterpenes (52.54%), monoterpenes (18.84%), and non-terpenoid chemicals (23.36%) [11]. The results of FASE, which contains a higher number of unique chemicals, including *β*-amyrin, which displayed improved bioactivity in multiple bioassays [12], are consistent. Several studies have found that bioactive substances, including dimethyl-2,3,4,7,8,9,11,12,14,15,16,17-dodecahydro-*1H*-cyclopenta[a]phenanthren-3-ol and (*1R*,*3aR*,*5aR*,*5bR*,*7aR*,*9S*,*11aR*,*11bR*,*13aR*,*13bR*)-3a,5a,5b,8,8,11a-Hexamethyl-1-(prop-1-en-2-yl)-1,2,3,4,5,6,7,7a,9,10,11,11b,12,13,13a,13b-hexadecahydrocyclopenta[a]chrysen-9-ol, have anticancer and anti-inflammatory properties [13,14,15,16].

TPC and TFC of FALE and FASE were determined to be 95.18 ± 1.67 mg GAE/g and 56.19 ± 1.94 mg QE/g for leaves, and 104.13 ± 1.25 mg GAE/g and 62.16 ± 1.34 mg QE/g for seeds, respectively. FASE contains more phenolics and flavonoids than FALE. The results were also compared with the values for the previously reported methanolic extract of *F. arabica* leaves, which showed a TPC of 867.7 ± 273.29 mg GAEs/g DW and a TFC of 70.7 ± 2.31 mg QUEs/g DW [17]. Another study reported a TPC value of 47.4 ± 5.1 mg GAEs/g DW for the herbal extract of the Indian *F. arabica* [18]. Walbi et al. (2023) reported low TPC and TFC values (1.3 mg GAE/g DW and 0.5 mg QE/g DW, respectively) for the methanolic extract of the whole *F. arabica* plant, highlighting the effects of differences in solvent extraction methods [19]. The TPC values for *F. arabica* fall within the range reported for other *Zygophyllaceae* species. For example, the stems of *Z. paulayanum* are reported to be rich in kaempferol derivatives and to show strong antioxidant activity [20]. Similarly, methanol extracts and all fractions of the root and aerial parts of *F. cretica* were reported to have strong antioxidant activities, attributed to their high TPC and TFC values, ranging from 0.23-4.30 mg/L GAE and from 30-545 mg/L RE, respectively [5]. The highest TPC and TFC in FASE would justify its later antioxidant, antimicrobial, anticancer, and antidiabetic activities. These activities are attributed to phenolics and flavonoids, which play a vital role in scavenging free radicals and modifying key biological pathways.

The free radical scavenging activities of FALE and FASE were determined by DPPH and ABTS^+^ assays and expressed as IC_50_ values. Both compounds exhibited significant, concentration-dependent free radical scavenging activity. DPPH assay IC_50_ values for FALE and FASE were found to be 78.2 and 63.3 μg/mL, while ABTS^+^ assay IC_50_ values for FALE and FASE were found to be 57.4 and 42.4 μg/mL, respectively. The ABTS^+^ assay IC_50_ value for FASE (42.4 μg/mL) was comparable to that of ascorbic acid used as a positive control in this study (41.2 μg/mL). The DPPH assay IC_50_ values for FASE and FALE were in good agreement with previously reported data. The DPPH assay IC_50_ value for the aerial parts of *F. arabica* collected from Egypt was 58.62 mg/L (approximately 58.6 μg/mL), which is close to the FASE value (63.3 μg/mL) in this study [21]. Another Egyptian study reported the value of 46.25 μg/mL for the DPPH assay of the aerial flowering parts of *F. arabica* using the aqueous extraction method [3]. This value is lower than the present study’s FASEvalue (63.3 μg/mL). The different extraction methods and the phenological stages of the plant under study could be the reasons for such variation. The aerial parts of *F. arabica* have been well established for their antioxidant activities, which are attributed to the high polyphenolic content in the plant [2]. The results of the present study showed that FASE exhibited stronger ABTS^+^ scavenging activities than FALE, which were correlated with their TPC and TFC.

In contrast, ABTS^+^ scavenging activity of FASE was found to be higher than that of FALE, and this can be correlated with higher TPC and TFC of FASE. Other members of *Zygophyllaceae*, such as *Z. paulayanum*, *Z. coccineum*, and *Tribulus terrestrii* have been reported for their excellent antioxidant activities [11,22,23]. It is possible to correlate the radical scavenging activities of FASE with the presence of some of the bioactive compounds such as (*9Z*,*12Z*,*15Z*)-octadeca-9,12,15-trienoic acid, (*3S*,*4aR*,*6aR*,*6bS*,*8aR*,*12aR*,*14aR*,*14bR*)-4,4,6a,6b,8a,11,11,14b-octamethyl-1,2,3,4a,5,6,7,8,9,10,12,12a,14,14a-tetradecahydropicen-3-ol, and (*1R*,*3aR*,*5aR*,*5bR*,*7aR*,*9S*,*11aR*,*11bR*,*13aR*,*13bR*)-3a,5a,5b,8,8,11a-Hexamethyl-1-(prop-1-en-2-yl)-1,2,3,4,5,6,7,7a,9,10,11,11b,12,13,13a,13b-hexadecahydrocyclopenta[a]chrysen-9-ol. A previous study evaluated bioactive constituents and medicinal values of some Saudi Arabian medicinal plants from the Tabuk region and showed that the aerial parts of *F. arbica* collected from Jabal-al-Lawz showed higher levels of bioactive compounds than those collected from Wadi-el-Dissa, which contributed to their high antioxidant activities [24]. So, the results of the present study, which investigated the antioxidant activities of *F. arabica* collected from Riyadh, are in good agreement with previous reports on the high antioxidant activity of Saudi Arabian *F. arabica*.

Regarding antimicrobial activity, all tested strains showed antibacterial and antifungal activity by FASE and FALE; however, FASE was more active than FALE across all strains. The MIC of FASE extract on *S. aureus* and *E. faecalis* was 7.81 µg/mL and 15.63 µg/mL, respectively. The disk diffusion assay demonstrated that FASE produced a ZoI of 21 ± 1 mm against *S. aureus* at 800 µg/mL, which was comparable to that of the positive control, chloramphenicol (22 ± 1 mm). For *E. faecalis*, ZoI values were 22 ± 2 mm (chloramphenicol, 23 ± 1 mm), and for *E. coli*, they were 17 ± 1 mm (positive control, chloramphenicol (20±1 mm). Moreover, the MIC values for all three *Candida* species tested by FASE were 15.63 µg/mL. These results are comparable with the previous literature. In an Egyptian study by Mahmoud et al. (2025), the flower extract of *F. arabica* showed significant ZOIs against *S. epidermidis* (20 mm), *S. aureus* (22 mm), *E. faecalis* (23 mm), *E. coli* (21 mm), *K. pneumoniae* (17 mm), and *Acinetobacter baumannii* (18 mm) [3]. In traditional Arabic medicine, *F. arabica* has been used as an antimicrobial for sore mouth and smallpox [2]. The results from the FASE in this work showed that it had good antifungal activity, with a ZoI of 20 ± 2 mm at 800 µg/mL against *C. parapsilosis*. These species and other related *Candida* species are opportunistic pathogens that are increasingly resistant to available antifungal agents. Compared with other *Zygophyllaceae* species, a previous study reported the antimicrobial activities of kaempferol derivatives from *Z. paulayanum* [20]. Another study reported the antibacterial activities of the methanolic extract of *F. indica* (*Zygophyllaceae*) from the Hail Mountains, Saudi Arabia, against *B. subtilis* (15 mm) and *P. aeruginosa* (12 mm) [6]. FASE displayed high antimicrobial activities, which can be correlated with the presence of anthraquinones (danthron and alizarin) and pyranones, as these compounds may disrupt microbial cell membranes and may also be able to inhibit microbial enzyme activities [25,26]. So, FASE could be exploited as a source of antimicrobial agents, especially amid current challenges of high antibiotic resistance.

The MTT assay results indicated concentration-dependent lethal effects of FALE and FASE against MCF-7 (breast) and HepG2 (liver) cancer cell lines. At 400 µg/mL, FASE significantly reduced MCF-7 cell viability to 23.29 ± 1.45% and HepG2 viability to 13.42 ± 1.14%, indicating superior activity, while the calculated IC_50_ values indicate moderate to strong cytotoxicity. qRT-PCR analysis showed that both extracts increased pro-apoptotic markers (caspase-3, 8, 9, and Bax) and decreased anti-apoptotic markers (Bcl-2 and Bcl-xL). These results are consistent with prior anticancer investigations on *F. arabica*. The Egyptian study by Mahmoud et al. (2025) showed mild cytotoxic activity of the flowering parts of *F. arabica* against the A549 lung cancer cell line (IC_50_ = 178.08 µg/mL) [3]. Another study reported cytotoxic activity of *F. arabica* against MCF-7 (breast), KB-3-1 (oral), and A549 (lung) cancer cells, with calculated IC_50_ values of 135.02 µg/mL, 195.21 µg/mL, and 116.06 µg/mL, respectively [19]. In the preliminary study by Sher et al. (2025), the methanolic extract of *F. arabica* leaves exhibited significant cytotoxicity against MCF-7 cells, with an IC_50_ of 181 µg/mL, accompanied by upregulation of caspases and downregulation of the Wnt/*β*-catenin signaling pathway [27]. Furthermore, the ethanolic extract of the aerial parts of the Egyptian *F. arabica* revealed cytotoxic activities against HepG2 (IC_50_ ~ 6.9 µg/mL), MCF-7 (IC_50_ ~ 7.6 µg/mL), and the intestinal CACO2 (IC_50_ ~ 9.2 µg/mL) cancer cells, with a 5.66-fold increase in caspase 9 expression [28]. Previous literature on the genus *Fagonia* confirms the cytotoxic and anticancer properties of several species, such as *F. indica*, against MCF-7 [29], MDA-MB-468, and CACO2 [30]. The present study is among the first to demonstrate that FASE is more potent than FALE against both breast and liver cancer cells. This finding is likely attributable to the higher triterpenoid content, including dimethyl-2,3,4,7,8,9,11,12,14,15,16,17-dodecahydro-*1H*-cyclopenta[a]phenanthren-3-ol, (*1R*,*3aR*,*5aR*,*5bR*,*7aR*,*9S*,*11aR*,*11bR*,*13aR*,*13bR*)-3a,5a,5b,8,8,11a-Hexamethyl-1-(prop-1-en-2-yl)-1,2,3,4,5,6,7,7a,9,10,11,11b,12,13,13a,13b-hexadecahydrocyclopenta[a]chrysen-9-ol, and (*3S*,*4aR*,*6aR*,*6bS*,*8aR*,*12aR*,*14aR*,*14bR*)-4,4,6a,6b,8a,11,11,14b-octamethyl-1,2,3,4a,5,6,7,8,9,10,12,12a,14,14a-tetradecahydropicen-3-ol, all of which have documented anticancer activities via modulation of apoptotic pathways [13,31,32,33]. Activation of caspase-8 (extrinsic pathway) and caspase-9 (intrinsic pathway) indicates that *F. arabica* extracts trigger apoptosis through multiple mechanisms, including death receptor ligation and mitochondrial dysfunction. Downregulation of Bcl-2 and Bcl-xL indicates participation of the intrinsic pathway. This complex approach is useful for overcoming chemoresistance and underscores FASE’s therapeutic potential as an adjuvant or standalone anticancer treatment. Future research should examine the seed extract’s efficacy and safety profile in animal models of breast and liver cancer. The inhibitory activities of FALE and FASE against major carbohydrate-digesting enzymes, α-amylases, and α-glucosidase were dose-dependent. Both enzymes were more effectively inhibited by FASE than by FALE, as indicated by their IC_50_ values. Importantly, the IC_50_ of FASE for α-glucosidase inhibition (67.38 µg/mL) was comparable to that of the well-known antidiabetic drug acarbose (66.13 µg/mL). Our results are consistent with previous studies reporting the antidiabetic activity of *Fagonia* species. In agreement with our findings, *F. arabica* crude extract and biosynthesized AgNPs showed α-glucosidase (IC_50_ = 92 µg/mL) and α-amylase (IC_50_ = 100 µg/mL) inhibition at 1 mg/mL and decreased blood glucose levels in streptozotocin (STZ)-treated diabetic rats [34]. These results were comparable and aligned with the antidiabetic activities of other *Fagonia* species. In a recent Turkish study, the methanolic seed and flower extracts of *F. indica* exhibited strong α-amylase inhibition (IC_50_ = 0.41 ng/µL), attributed to the antidiabetic phytochemical constituents chlorogenic acid, arbutin, isorhamnetin, and ursolic acid [35]. Another study reported the hypoglycemic effects of the water extract of *F. cretica* through *in vitro* α-glucosidase inhibition (IC_50_ = 4.62 µg/mL) and a 45% reduction in plasma glucose levels in STZ-treated diabetic rats [36]. The stronger inhibition of carbohydrate-digesting enzymes by FASE compared to FALE is associated with the levels of phenolic and flavonoid constituents. The active constituents of FASE, such as dimethyl-2,3,4,7,8,9,11,12,14,15,16,17-dodecahydro-*1H*-cyclopenta[a]phenanthren-3-ol and lupeol, have been reported to modulate glucose metabolism and insulin signaling [16,37]. In addition, the polyunsaturated fatty acid (*9Z*,*12Z*,*15Z*)-octadeca-9,12,15-trienoic acid has been shown to improve insulin sensitivity [38]. In line with the established α-amylase inhibitor activity and phenolic profile of the other members of the *Zygophyllaceae* family, the present findings suggest that the seeds of *F. arabica* can be exploited as a functional food or as a nutraceutical for the management of type 2 diabetes.

The study presents additional data on *F. arabica*, a plant species studied for the first time in Riyadh, Kingdom of Saudi Arabia, with respect to its leaves and seeds. The majority of studies on *Fagonia* species have focused on the phytochemical constituents of the aerial parts or the whole plant. In the current study, new insights into the phytochemical composition of the seed extract highlighted important steroidal derivatives, *β*-amyrin, and dihomo-*γ*-linolenic acid, which were reported for the first time from *F. arabica*. The study demonstrated that the seed extract exhibited higher biological activity than the leaf extract across all five bioassays (antioxidant, antibacterial, antifungal, anticancer, and antidiabetic). The study also provided new insights into the mechanism of action of FASE -induced apoptosis in two human cancer cell lines, HepG2 and MCF-7, through activation of both the intrinsic (*caspase-9* and *Bax*) and extrinsic (*caspase-8*) pathways. Finally, the study contributes to the growing body of evidence on the pharmacological activities of the Saudi Arabian medicinal flora. Although this study presents robust data with a wide array of results and data to support the study’s findings, some limitations should be noted. For example, the current study is an *in vitro*-based study; therefore, *in vitro* data cannot be used to establish the *in vivo* efficacy or safety of the tested extracts. Also, we recognize that it was a tentative identification of phytochemicals without using authentic standards, that require further investigations for identifying semivolatile and non-volatile compounds.

As noted above, the pharmacokinetics of a compound and its bioavailability can dramatically affect the activity and bioeffects of a phytochemical on human health. For cytotoxicity assays, only two cancer cell lines, MCF-7 and HepG2, were tested, and therefore, the selectivity index of the extracts (i.e., cytotoxicity to cancer cells vs. normal cells) is currently unknown. In addition, the GC–MS-identified compounds were only identified by matching the mass spectra to entries in the NIST and Wiley libraries. However, in the absence of any available standards or isolated compounds, some of the identified compounds are only tentative. In addition, the study reports only the bioeffects of the tested extracts on the inhibition of enzymes associated with diabetes; therefore, these data should be supported by *in vivo* experiments to evaluate glucose uptake regulation in animal models of diabetes, and it is also important to investigate possible side effects of such inhibition.

## 4. Materials and Methods

### 4.1. Preparation of Aqueous Methanolic Extracts from Leaves and Seeds of F. arabica

Whole plants of *F. arabica* L. were collected during December 2025 from natural populations in Riyadh, Saudi Arabia. The species was identified on the basis of its diagnostic morphological characteristics using the taxonomic keys provided in the Flora of the Kingdom of Saudi Arabia [39]. Plant authentication was performed by specialists at the Herbarium of the Department of Botany and Microbiology, College of Science, King Saud University (Riyadh, Saudi Arabia), where a voucher specimen (No. KSU NO-103415) has been deposited for future reference.

After collection, the plant material was washed thoroughly with distilled water to remove adhering soil and debris, shade-dried under ambient laboratory conditions (25 ± 2 °C) with adequate ventilation until a constant weight was obtained, and stored in clean polyethylene bags before extraction. The air-dried leaves and seeds were milled separately into a homogeneous powder using a Pulverisette 14 mill (Fritsch GmbH, Idar-Oberstein, Germany) fitted with a 0.5-mm sieve. For each extraction, 20 g of powdered material was mixed with 100 mL of 80% (*v*/*v*) methanol and extracted by maceration at 25 ± 2 °C for 48 h with continuous orbital shaking at 150 rpm using an IKA KS 4000 i control incubator shaker (IKA-Werke GmbH & Co. KG, Staufen im Breisgau, Germany). The extraction was performed in three consecutive cycles, after which the filtrates were pooled, passed through Whatman No. 1 filter paper, and concentrated under reduced pressure at 40 °C using a Rotavapor R-200 (Büchi Labortechnik, Flawil, Switzerland). The concentrated extracts were dried to constant weight, stored at −20 °C, and reconstituted immediately before analysis in the appropriate solvent. HPLC-grade methanol was used for GC–MS analysis, whereas 0.1% (*v*/*v*) DMSO served as the solvent for all biological assays. The dried plant materials were pulverized into a fine powder using a Pulverisette 14 mill (Fritsch GmbH, Idar-Oberstein-Georg-Weierbach, Germany) equipped with a 0.5 mm sieve. The extraction yield was calculated using the following equation [40]:

Yield (%) = weight of solvent-free extract (g) × 100/dried extract weight(1)

### 4.2. Analysis of Phytochemicals in FALE and FASE

The phytochemical composition of the FALE and FASE was characterized using an Agilent 7890B gas chromatograph coupled to an Agilent 5977A mass selective detector (Agilent Technologies, Santa Clara, CA, USA). Chromatographic separation was achieved on a DB-5MS fused-silica capillary column (30 m × 0.25 mm i.d., 0.25 μm film thickness). Helium (99.999% purity) served as the carrier gas at a constant flow rate of 1.0 mL/min. Aliquots (1.5 μL) of the methanolic extracts were introduced automatically in split injection mode (1:10), with the injector maintained at 250 °C.

The GC oven was operated using a programmed temperature gradient from 50 °C to 250 °C, giving a total analytical run time of 73 min. Mass spectral data were acquired under electron ionization (EI) at 70 eV. The ion source, quadrupole, and transfer line temperatures were maintained at 230 °C, 150 °C, and 280 °C, respectively. A 2 min solvent delay was applied before data acquisition, and ions were monitored over an m/z range of 40–500. Identification of the detected constituents was achieved by comparing the acquired mass spectra with reference spectra available in the National Institute of Standards and Technology (NIST) and Wiley mass spectral libraries. Only compounds exhibiting a spectral matching score exceeding 90% were accepted as positively identified.

Each extract underwent a single GC-MS analysis. The extracts examined were pooled samples from three successive maceration cycles. Future investigations will include technical replicates to ensure quantitative validity.

### 4.3. Determination of TPC and TFC

The TPC and TFC of the methanolic FALE and FASE were quantified by spectrophotometric assays based on previously established protocols with slight methodological modifications. TPC was estimated using the Folin–Ciocalteu colorimetric assay [41]. Each extract was prepared at a concentration of 1 mg/mL, and 100 μL was transferred into a reaction tube containing distilled water and Folin–Ciocalteu reagent. After thorough mixing, 20% (*w*/*v*) sodium carbonate solution was added to develop the characteristic blue chromophore. The mixtures were maintained at ambient temperature in the absence of light for 20–30 min before absorbance was measured at 765 nm using a Multiskan Sky microplate spectrophotometer (Thermo Fisher Scientific, Waltham, MA, USA). A calibration curve generated from gallic acid (20–200 μg/mL) was used for quantification, and the results were expressed as mg gallic acid equivalents (GAE) per gram of dry extract (DW).

TFC was evaluated independently using the aluminum chloride complexation assay described by Ordonez et al. [42]. Briefly, 100 μL of each extract was combined with 3.0 mL of 2% (*w*/*v*) aluminum chloride solution and allowed to react for 30 min at room temperature. Following incubation, absorbance was recorded at 420 nm with the same instrument. Quantification was achieved using a quercetin standard curve (20–200 μg/mL), and the total flavonoid content was reported as mg quercetin equivalents (QE) per gram of dry extract (DW).

### 4.4. Assessment of Antioxidant Activity in FALE and FASE

The antioxidant potential of the FALE and FASE was assessed using two complementary free radical scavenging assays, namely DPPH and ABTS, following published procedures with slight modifications [43,44]. For the DPPH assay, methanolic solutions of the extracts were prepared at concentrations ranging from 25 to 200 μg/mL. An aliquot of 200 μL of each concentration was mixed with 2.0 mL of freshly prepared 0.08 mM DPPH solution in methanol. The reaction mixtures were protected from light and maintained at room temperature for 20 min before absorbance was measured at 517 nm using a Thermo Scientific Multiskan Sky spectrophotometer (Thermo Fisher Scientific, Waltham, MA, USA).

The ABTS assay was performed independently. The radical solution was generated by reacting 7 mM ABTS with 2.45 mM potassium persulfate, followed by incubation in the dark at 28 ± 1 °C for 12 h. Prior to use, the solution was diluted with ethanol until its absorbance reached 0.50–0.60 at 734 nm. Subsequently, 25 μL of each extract solution (12.5–100 μg/mL) was added to 1.925 mL of the ABTS working solution and allowed to react in the dark at 28 °C for 20 min. The absorbance was then recorded at 734 nm using the same spectrophotometer. The ascorbic acid was served as the positive antioxidant control in both the DPPH and ABTS assays. Radical scavenging capacity was quantified as the percentage reduction in absorbance compared with the untreated control. Antioxidant potency was further evaluated by calculating the IC_50_ values, corresponding to the concentration of extract necessary to achieve 50% inhibition of free radicals. An IC_50_ values were obtained by nonlinear curve fitting using GraphPad Prism version 5.0 (GraphPad Software Inc., La Jolla, CA, USA).

### 4.5. Cell Culture, Cytotoxicity, and Gene Expression Analysis

The cytotoxic and apoptosis-inducing activities of FAL and FAS extracts were evaluated using MCF-7 (ATCC HTB-22) and HepG2 (ATCC HB-8065) cell lines (ATCC, USA). Cells at passages 12–15 were cultured in DMEM supplemented with 10% fetal bovine serum, 2 mM L-glutamine, and 1% penicillin–streptomycin, and maintained at 37 °C in a humidified incubator with 5% CO_2_. For the MTT assay, cells (1 × 10^4^ cells/well) were seeded into 96-well plates, incubated for 24 h, and treated with FALE or FASE (50–400 μg/mL) for 24–48 h. Untreated cells and cisplatin (30 μg/mL) served as negative and positive controls, respectively. Cell viability was determined by the MTT assay, and IC_50_ values were calculated using GraphPad Prism version 5.0 (GraphPad Software Inc., La Jolla, CA, USA). For apoptosis analysis, cells were exposed to the respective IC_50_ concentrations of each extract for 48 h. Total RNA was extracted using the RNeasy Kit (Qiagen, Hilden, Germany), and the expression of caspase-3, caspase-8, caspase-9, Bax, Bcl-2, and Bcl-xL was quantified by qRT-PCR following Aziz et al. [45]. Gene expression levels were normalized to GAPDH and calculated using the 2^−ΔΔCq^ method [46].

### 4.6. Screening for Antimicrobial Activity

FALE and FASE were subjected to antimicrobial activity screening against various Gram-positive and Gram-negative bacteria as well as some pathogenic fungi. Selected bacteria were *S. aureus* ATCC 29213, *E. faecalis* ATCC 29212, and *B. subtilis* ATCC 23857 as Gram-positive; *E. coli* ATCC 25922, *K. pneumoniae* ATCC 13883, and *P. aeruginosa* ATCC 27853 as Gram-negative. Selected pathogenic fungi were *C. albicans* ATCC 10231, *C. glabrata* ATCC 2001, and *C. parapsilosis* ATCC 22019.

Standardized microbial suspensions (0.5 McFarland) in Mueller-Hinton broth for bacteria and Sabouraud dextrose broth for fungi were prepared. The disc diffusion assay was performed with 6 mm sterile filter paper discs (Cytiva, Marlborough, MA, USA) containing 10 μL each of FALE or FASE (125, 250, 500, and 1000 μg/mL) [47,48]. Chloramphenicol and fluconazole (25 μg/mL) were used as positive controls for antibacterial and antifungal assays, respectively. The negative control was 0.1% DMSO. Following diffusion at 4 °C for 2 h, bacterial (fungal) plates were incubated at 37 °C (28 °C) for 24 h (72 h). The activity was designated as the ZoI. The broth microdilution assay was used next to determine the MIC, MBC, and MFC [49,50,51]. The extracts were serially diluted (1.95–1000 μg/mL) into the appropriate broth media in 96-well plates and inoculated (approximately 1 × 10^2^ CFU/mL). The plates were incubated for 24 h. The colored 2,3,5-triphenyl tetrazolium chloride (TTC) (20 μL of 2 mg/mL) was added to each well. The MIC was recorded as the lowest concentration where no color developed. The MBC and MFC were the lowest concentrations of a color reaction, following a subculture onto solid media, to inhibit microbial growth.

### 4.7. α-Amylase and α-Glucosidase Inhibitory Assays

Using acarbose as the reference inhibitor, the antidiabetic potential of FALE and FASE was assessed by measuring their inhibitory activities against α-amylase and α-glucosidase. With a few minor modifications, the α-amylase assay was carried out using the DNSA method [52]. After incubating extracts (25–400 μg/mL) or the positive control (acarbose) in phosphate buffer (pH 6.9) with α-amylase (2 U/mL), 1% starch was added as the substrate. Following incubation, the reaction was stopped with DNSA reagent, heated to 85 °C for 10 min, diluted with distilled water, and the absorbance was measured at 540 nm using a Thermo Scientific Multiskan Sky spectro-photometer (Thermo Fisher Scientific, Waltham, MA, USA).

According to Kim et al. [53], α-glucosidase inhibition was measured using yeast α-glucosidase (1 U/mL) and p-nitrophenyl-α-D-glucopyranoside (pNPG) as the substrate. After the enzyme was incubated with the extracts (25–400 μg/mL) or the positive control (acarbose), pNPG was used to start the reaction, 1 M sodium carbonate was used to stop it, and the same device was used to measure absorbance at 405 nm. Reaction mixtures devoid of extract were used as negative controls for both tests. The percentage inhibition in relation to the control was used to express enzyme inhibition, and GraphPad Prism version 5.0 (GraphPad Software Inc., La Jolla, CA, USA) was used to calculate IC_50_ values by nonlinear regression.

### 4.8. Statistical Analysis

Each experiment was carried out in triplicate, and the results are shown as the mean ± SD. IBM SPSS Statistics and GraphPad Prism version 5.0 (GraphPad Software Inc., La Jolla, CA, USA) were used for the statistical analyses. The Mann–Whitney U test or the unpaired Student’s *t*-test were used to assess differences between two groups, and one-way ANOVA and the least significant difference (LSD) post hoc test were used to examine multiple comparisons. To guarantee data reliability, experiments for cytotoxicity, antioxidant, antimicrobial, anticancer, and gene expression were independently repeated three times. The threshold for statistical significance was set at *p* < 0.05.

## 5. Conclusions

In the present study, FALE and FASE were found to contain pharmacologically active constituents. FASE had the highest TPC and TFC. Antioxidant activity was potent and dose-dependent. Antimicrobial activity was broad-spectrum, with low MIC values against S. aureus and *Candida* spp., comparable to those of conventional antibiotics. In addition to these activities, the extracts showed potent anticancer activity against the human cancer cell lines MCF-7 and HepG2 and significant antidiabetic activity through inhibition of the enzymes α-amylase and α-glucosidase. In summary, these extracts contain a novel, multitargeted natural product with great therapeutic potential for treating diseases caused by oxidative stress, as well as various infectious diseases, cancer, and diabetes. Further *in vivo* studies, bioassay-guided fractionation, and clinical trials are required to translate the findings of the present study into practical applications.

## Figures and Tables

**Table 1:** GC–MS compounds in FALE.

Peak	Rt	Area	Area%	Name	MF	MW	CAS No.	Class
1	8.282	39,738,877	0.81	2,6-dimethylocta-2,6-diene-1,8-diol	C_10_H_18_O_2_	170	692-06-8	Monoterpenoid
2	10.261	205,332,961	4.21	2-methylpentanoic acid	C_6_H_12_O_2_	116	97-61-0	Branched-chain fatty acid
3	12.595	622,386,734	12.75	3,5-dihydroxy-6-methyl-2,3-dihydro-4H-pyran-4-one	C_6_H_8_O_4_	144	28564-83-2	Pyranone
4	12.813	81,317,692	1.66	5-methyl-2-propan-2-ylcyclohexan-1-ol	C_10_H_20_O	156	2216-51-5	Monoterpenoid
5	13.981	9,258,486	0.18	3,5-dihydroxy-2-methylpyran-4-one	C_6_H_6_O_4_	142	1073-26-3	Pyranone
6	15.289	14,882,379	0.31	5-(hydroxymethyl)furan-2-carbaldehyde	C_6_H_6_O_3_	126	67-47-0	Furanic aldehyde
7	16.009	16,601,719	0.34	3,7-dimethyloct-6-enal	C_10_H_18_O	154	106-23-0	Monoterpenoid
8	16.553	81,351,167	1.67	4-ethenyl-2-methoxyphenol	C_9_H_10_O_2_	150	7786-61-0	Phenylpropanoid
9	16.791	9,748,932	0.2	1-methyl-5-(propan-2-yl)-3,6,7-trioxatricyclo[3.2.2.0(2,4)]nonane	C_10_H_16_O_3_	184	135760-25-7	Monoterpenoid
10	18.14	9,579,034	0.19	Methyl 4-methoxybenzoate	C_9_H_10_O_3_	166	121-98-2	Benzenoid ester
11	18.613	56,651,345	1.16	(*2Z*)-2-(3,3-dimethyl-2-bicyclo[2.2.1]heptanylidene)ethanol	C_11_H_18_O	166	2226-05-3	Monoterpenoid
12	19.031	15,750,975	0.32	2-[(*Z*)-2-methoxyprop-1-enyl]-1,3,5-trimethylbenzene	C_13_H_18_O	190	Not found	Benzene derivative
13	19.191	14,039,502	0.29	methyl 3,5-dioxo-2,6,7,8-tetrahydro-1H-pyrrolizine-2-carboxylate	C_9_H_11_NO_4_	197	Not found	Pyrrolizidine alkaloid derivative
14	19.903	11,174,571	0.23	3-Phenylprop-2-en-1-yl acetate	C_11_H_12_O_2_	176	103-54-8	Phenylpropanoid ester
15	20.473	9,994,772	0.21	(*E*)-4-(2,6,6-trimethylcyclohex-2-en-1-yl)but-3-en-2-one	C_13_H_20_O	192	127-41-3	Norisoprenoid
16	20.741	6,928,392	0.14	2,2,6,7-tetramethyl-10-oxatricyclo[5.2.1.01,6]decan-5-ol	C_13_H_22_O_2_	210	Not found	Sesquiterpenoid
17	20.894	5,284,462	0.11	5-hydroxy-9-oxabicyclo[3.3.1]nonan-2-one	C_8_H_12_O_3_	156	Not found	Lactone
18	21.109	2,552,565	0.05	(−)-dihydrocarvyl ethanoate	C_12_H_20_O_2_	196	18697-98-8	Monoterpenoid
19	21.764	87,466,228	1.79	1-methyl-4-propan-2-ylcyclohexane-1,2,3-triol	C_10_H_20_O_3_	188	18973-00-7	Monoterpenoid
20	21.928	11,889,379	0.24	(*4aR*,*8aR*)-8a-hydroxy-4a-methyl-3,4,4a,5,6,7,8,8a-octahydronaphthalen-2(1H)-one	C_11_H_18_O_2_	182	Not found	Sesquiterpenoid
21	22.056	23,399,926	0.48	2,5,5,8a-tetramethyl-5,6,7,8-tetrahydro-*8H*-chromen-8-ol	C_13_H_20_O_2_	208	97306-62-2	Chromene derivative
22	22.58	11,840,128	0.24	5-[(*Z*)-oct-1-enyl]oxolan-2-one	C_12_H_20_O_2_	196	58249-55-1	Unsaturated lactone
23	23.007	27,630,112	0.56	1-methoxy-4,4a,5,6,7,8-hexahydro-3H-naphthalen-2-one	C_11_H_16_O_2_	180	Not found	Sesquiterpenoid
24	23.249	7,347,850	0.15	(*3R*,*9Z*)-heptadeca-1,9-dien-4,6-diyn-3-ol	C_17_H_24_O	244	21852-80-2	Polyacetylene alcohol
25	23.428	31,556,737	0.65	dodecanoic acid	C_12_H_24_O_2_	200	143-07-7	Saturated fatty acid
26	25.283	14,089,423	0.29	2-(4-hydroxy-3-methoxyphenyl)acetic acid	C_9_H_10_O_4_	182	306-08-1	Phenolic acid
27	26.378	11,449,623	0.23	2,5,9,9-tetramethyl-5,8-dihydrobenzo[7]annulene	C_15_H_20_	200	51766-65-5	Sesquiterpene
28	26.561	16,483,763	0.38	3,8-dimethyl-5-propan-2-yl-1,2-dihydronaphthalene	C_15_H_20_	200	20129-39-9	Sesquiterpene
29	26.776	95,661,411	1.96	2,2,7,7-tetramethyltricyclo[6.2.1.0^1,6]undeca-3,5,9-triene	C_15_H_20_	200	156747-45-4	Sesquiterpene
30	27.166	20,724,035	0.42	4,6,10,10-tetramethyl-5-oxatricyclo[4.4.0.01,4]dec-2-en-7-ol	C_13_H_20_O_2_	208	Not found	Sesquiterpenoid
31	27.533	81,465,362	1.67	(*2E*)-2-(hexa-2,4-diynylidene)-1,6-dioxaspiro[4.4]non-3-ene	C_13_H_12_O_2_	200	13018-10-5	Polyacetylene spiroketal
32	27.927	32,226,025	0.66	tetradecanoic acid	C_14_H_28_O_2_	228	544-63-8	Saturated fatty acid
33	28.06	10,664,595	0.22	(*1S*,*4aS*,*8aS*)-[(2,5,5,8a-tetramethyl-1,4,4a,6,7,8-hexahydronaphthalen-1-yl)]methanol	C_15_H_26_O	222	468-68-8	Sesquiterpenoid
34	28.181	16,320,180	0.33	(*4S*)-4-hydroxy-3,5,5-trimethyl-4-[(*E*)-3-oxobut-1-enyl]cyclohex-2-en-1-one	C_13_H_18_O_3_	222	15764-81-5	Norisoprenoid
35	28.971	16,352,067	0.33	pentadecanal	C_15_H_30_O	226	2765-11-9	Long-chain aldehyde
36	29.117	10,639,639	0.22	6,10,14-trimethylpentadecan-2-one	C_18_H_36_O	268	502-69-2	Ketone
37	29.495	13,607,410	0.28	7,11,15-trimethyl-3-methylidenehexadec-1-ene	C_20_H_38_	278	504-96-1	Diterpene hydrocarbon
38	29.999	18,773,848	0.38	pentadecanoic acid	C_15_H_30_O_2_	242	1002-84-2	Saturated odd-chain fatty acid
39	30.187	32,380,839	0.66	(*E*)-4-(3-hydroxy-2,6,6-trimethylcyclohex-1-en-1-yl)but-3-en-2-one	C_13_H_20_O_2_	208	25312-47-8	Norisoprenoid
40	30.396	16,372,781	0.33	hexadeca-7,11-dienal	C_16_H_28_O	236	Not found	Unsaturated long-chain aldehyde
41	30.521	32,326,403	0.66	(*1R*,*2S*,*7S*,*8S*,*9R*)-2,6,6,9-tetramethyltricyclo[5.4.0.0^2,9]undecan-8-ol	C_15_H_26_O	222	465-24-7	Sesquiterpenoid
42	31.259	12,465,696	0.26	(*3aS*,*5aR*,*6R*,*9aS*,*9bS*)-6-hydroxy-5a,9-dimethyl-3-methylidene-4,5,6,7,9a,9b-hexahydro-3aH-benzo[g][1]benzofuran-2-one	C_15_H_20_O_3_	248	4290-13-5	Sesquiterpene lactones
43	31.457	15,545,751	0.32	(*E*)-hexadec-2-enal	C_16_H_30_O	238	5910-29-2	Unsaturated aldehyde
44	31.601	19,217,948	0.39	(*Z*)-14-methylhexadec-8-enal	C_17_H_32_O	252	60609-53-2	Branched unsaturated aldehyde
45	32.536	613,934,628	12.57	hexadecanoic acid	C_16_H_32_O_2_	256	57-10-3	Saturated fatty acid
46	34.269	64,908,605	1.33	(*E*,*7R*,*11R*)-3,7,11,15-tetramethylhexadec-2-en-1-ol	C_20_H_40_O	296	150-86-7	Diterpenoid
47	34.411	8,264,351	0.17	hexadec-9-enoic acid	C_16_H_30_O_2_	254	373-49-9	Monounsaturated fatty acid
48	34.527	32,456,727	0.66	eicosanal	C_20_H_40_O	296	2400-66-0	Long-chain aldehyde
49	35.95	694,459,376	14.22	(*9Z*,*12Z*,*15Z*)-octadeca-9,12,15-trienoic acid	C_18_H_30_O_2_	278	463-40-1	Linolenic/polyunsaturated fatty acid and derivatives
50	36.213	118,514,035	2.42	octadecanoic acid	C_18_H_36_O_2_	284	57-11-4	Saturated fatty acid
51	37.944	8,946,971	0.18	[(*2E*)-3,7-dimethylocta-2,6-dienyl] 3-methylbutanoate	C_15_H_26_O_2_	238	109-20-6	Monoterpenoid
52	38.277	8,111,592	0.17	3-hydroxydodecanoic acid	C_12_H_24_O_3_	216	1883-13-2	Hydroxy fatty acid
53	39.409	9,007,915	0.18	(*E*)-octadec-13-enoic acid	C_18_H_34_O_2_	282	13126-38-0	Trans-unsaturated fatty acid
54	39.849	89,901,480	1.84	1,8-dihydroxyanthracene-9,10-dione	C_14_H_8_O_4_	240	117-10-2	Anthraquinone
55	40.892	53,143,322	1.08	1,2-dihydroxyanthracene-9,10-dione	C_14_H_8_O_4_	240	72-48-0	Anthraquinone
56	41.332	27,655,319	0.56	icosan-1-ol	C_20_H_42_O	298	629-96-9	Long-chain fatty alcohol
57	41.982	73,256,780	1.51	1,3-dihydroxypropan-2-yl hexadecanoate	C_19_H_38_O_4_	330	23470-00-0	Monoacylglycerol
58	43.652	46,388,296	0.95	(*2E*,*6E*,*10E*)-3,7,11,15-tetramethylhexadeca-2,6,10,14-tetraen-1-ol	C_20_H_34_O	290	24034-73-9	Diterpenoid
59	45.692	71,875,114	1.47	[(*2S*)-2,3-dihydroxypropyl] (*9Z*,*12Z*,*15Z*)-octadeca-9,12,15-trienoate	C_21_H_36_O_4_	352	122-43-0	Linolenic/polyunsaturated fatty acid and derivatives
60	46.496	58,551,734	1.19	[(*2S*)-2,3-dihydroxypropyl] (*9Z*,*12Z*)-octadeca-9,12-dienoate	C_21_H_38_O_4_	354	3443-82-1	Monoacylglycerol

**Table 2:** GC–MS compounds in FASE.

Peak	Rt	Area	Area%	Name	MF	MW	CAS No.	Class
1	8.264	34,703,080	0.51	(*1R*,*6R*)-3-methyl-6-propan-2-ylcyclohex-2-en-1-ol	C_10_H_18_O	154	16721-39-4	Monoterpenoid
2	9.753	291,382,806	4.36	2-methylpentanoic acid	C_6_H_12_O_2_	116	97-61-0	Branched-chain fatty acid
3	10.667	897,526,106	13.43	2-methylhexanoic acid	C_7_H_14_O_2_	130	4536-23-6	Medium-chain fatty acid
4	12.531	509,913,859	7.63	3,5-Dihydroxy-6-methyl-2,3-dihydro-4H-pyran-4-one	C_6_H_8_O_4_	144	28564-83-2	Pyranone
5	13.877	11,285,720	0.16	3,5-dihydroxy-2-methylpyran-4-one	C_6_H_6_O_4_	142	1073-26-3	Pyranone
6	14.751	14,026,695	0.21	Bicyclo[3.3.1]non-2-en-9-ol	C_9_H_14_O	138	Not found	Cyclic alcohols and derivatives
7	14.971	9,673,541	0.14	4-methoxybenzaldehyde	C_8_H_8_O_2_	136	123-11-5	Aromatic aldehyde
8	15.269	178,843,089	2.67	5-(hydroxymethyl)furan-2-carbaldehyde	C_6_H_6_O_3_	126	67-47-0	Furanic aldehyde
9	16.004	17,076,906	0.26	3,7-dimethyloct-6-enal	C_10_H_18_O	154	106-23-0	Monoterpenoid
10	16.89	8,265,247	0.12	4-ethenyl-2-methoxyphenol	C_9_H_10_O_2_	150	7786-61-0	Methoxyphenols
11	17.176	13,874,023	0.21	3-methyl-1-(2,4,6-trimethylphenyl)butan-1-ol	C_14_H_22_O	206	Not found	Monoterpenoids
12	18.215	45,584,056	0.68	methyl 4-methoxybenzoate	C_9_H_10_O_3_	166	121-98-2	Methoxybenzoic acids and derivatives
13	18.633	72,283,902	1.08	(*2Z*)-2-(3,3-dimethyl-2-bicyclo[2.2.1]heptanylidene)ethanol	C_11_H_18_O	166	2226-05-3	Monoterpenoids
14	19.039	22,157,946	0.33	2-[(*Z*)-2-methoxyprop-1-enyl]-1,3,5-trimethylbenzene	C_13_H_18_O	190	Not found	Benzene and substituted derivatives
15	19.766	9,333,963	0.14	(*E*)-undec-2-enoic acid	C_11_H_20_O_2_	184	15790-73-5	Medium-chain fatty acids
16	20.172	23,227,970	0.35	5-hydroxy-9-oxabicyclo[3.3.1]nonan-2-one	C_8_H_12_O_3_	156	Not found	Lactones
17	20.464	22,597,730	0.34	(*E*)-4-(2,6,6-trimethylcyclohex-2-en-1-yl)but-3-en-2-one	C_13_H_20_O	192	127-41-3	Sesquiterpenoids
18	20.742	20,600,152	0.31	2-nonyl-2,3-dihydropyran-6-one	C_14_H_24_O_2_	224	22501-22-0	Pyranones and derivatives
19	20.937	6,299,958	0.09	cyclopentadecanone	C_15_H_28_O	224	502-72-7	Cyclic ketones
20	21.76	63,085,326	0.94	1-methyl-4-propan-2-ylcyclohexane-1,2,3-triol	C_10_H_20_O_3_	188	18973-00-7	Monoterpenoid
21	23.009	27,600,570	0.41	1-methoxy-4,4a,5,6,7,8-hexahydro-2(*3H*)-naphthalenone	C_11_H_16_O_2_	180	Not found	Cyclic ketones
22	23.368	42,932,600	0.64	(*3R*,*9Z*)-heptadeca-1,9-dien-4,6-diyn-3-ol	C_17_H_24_O	244	21852-80-2	Long-chain fatty alcohols
23	23.697	28,907,041	0.43	(*E*)-4-(4-hydroxy-2,6,6-trimethylcyclohex-2-en-1-yl)but-3-en-2-one	C_13_H_20_O_2_	208	215871-68-4	Sesquiterpenoids
24	24.165	10,755,289	0.16	2-methyl-5-prop-1-en-2-ylcyclopentene-1-carbaldehyde	C_10_H_14_O	150	3865-09-6	Monoterpenoid
25	24.328	7,090,454	0.11	4-[(*1E*)-buta-1,3-dienyl]-3,5,5-trimethylcyclohex-2-en-1-one	C_13_H_18_O	190	38818-55-2	Cyclic ketones
26	24.69	2,786,477	0.04	1,6,6-Trimethyl-7-[(***2Z***)-4-methylpenta-2,4-dien-2-yl]-3,8-dioxatricyclo[5.1.0.0(2,4)]octane	C_15_H_22_O_2_	234	Not found	Oxepanes
27	24.837	11,249,493	0.16	3,4,5-trimethoxyphenol	C_9_H_12_O_4_	184	642-71-7	Methoxyphenols
28	25.078	12,479,324	0.18	3,7,11-trimethyldodecan-1-ol	C_15_H_32_O	228	6750-34-1	Sesquiterpenoid
29	25.858	4,508,122	0.06	1-methyl-4-(6-methylhept-5-en-2-yl)benzene	C_15_H_22_	202	34143-96-9	Sesquiterpenoid
30	25.973	8,736,986	0.13	1-(6-Hydroxy-7-(propan-2-yl)-4-methylidene-2,3,3a,4,5,6,7,7a-octahydro-1H-inden-1-yl)ethanone	C_15_H_24_O_2_	236	Not found	Sesquiterpene
31	26.193	6,176,039	0.09	2,6-dimethoxy-4-prop-2-enylphenol	C_11_H_14_O_3_	194	55348-10-2	Methoxyphenols
32	26.358	12,787,930	0.19	2,5,9,9-tetramethyl-5,8-dihydrobenzo[7]annulene	C_15_H_20_	200	51766-65-5	Benzoids
33	26.538	20,047,502	0.31	3,8-dimethyl-5-propan-2-yl-1,2-dihydronaphthalene	C_15_H_20_	200	20129-39-9	Sesquiterpenoid
34	26.776	98,563,118	1.47	2,2,7,7-tetramethyltricyclo[6.2.1.01,6]undeca-3,5,9-triene	C_15_H_20_	200	156747-45-4	Polycyclic hydrocarbons
35	27.226	20,039,286	0.31	4,6,10,10-tetramethyl-5-oxatricyclo[4.4.0.01,4]dec-2-en-7-ol	C_13_H_20_O_2_	208	Not found	Pyrans
36	27.525	79,652,013	1.19	2,2-dimethyl-3-[(*Z*)-3-(4-methylcyclohex-3-en-1-yl)but-2-enyl]oxirane	C_15_H_24_O	220	Not found	Sesquiterpenoid
37	27.852	53,088,416	0.79	[(*1S*,*4aS*,*8aS*)-2,5,5,8a-tetramethyl-1,4,4a,6,7,8-hexahydronaphthalen-1-yl]methanol	C_15_H_26_O	222	468-68-8	Sesquiterpenoid
38	28.864	41,994,781	0.63	(*4S*)-4-hydroxy-3,5,5-trimethyl-4-[(*E*)-3-oxobut-1-enyl]cyclohex-2-en-1-one	C_13_H_18_O_3_	222	15764-81-5	Sesquiterpenoid
39	28.963	101,235,197	1.52	4a-hydroxy-4,8a-dimethyl-6-prop-1-en-2-yl-3,4,5,6,7,8-hexahydro-*2H*-naphthalen-1-one	C_15_H_24_O_2_	236	97094-19-4	Sesquiterpenoid
40	29.483	29,785,753	0.44	7,11,15-trimethyl-3-methylidenehexadec-1-ene	C_20_H_38_	278	504-96-1	Sesquiterpenoid
41	29.857	48,157,911	0.72	*4*,*4*,*7a*-trimethyl-*3a*,5,6,7-tetrahydro-*3H*-1-benzofuran-2-one	C_11_H_18_O_2_	182	17092-92-1	Benzofurans
42	30.197	30,662,507	0.46	(*E*)-4-(3-hydroxy-2,6,6-trimethylcyclohexen-1-yl)but-3-en-2-one	C_13_H_20_O_2_	208	25312-47-8	Sesquiterpenoids
43	30.491	21,508,994	0.32	hexadeca-7,11-dienal	C_16_H_28_O	236	Not found	Fatty aldehydes
44	30.731	22,612,968	0.34	(*8S*)-2,6,6,9-tetramethyltricyclo[5.4.0.02,9]undecan-8-ol	C_15_H_26_O	222	Not found	Monoterpenoids
45	31.339	150,270,509	2.25	(*3aS*,*5aR*,*6R*,*9aS*,*9bS*)-6-hydroxy-5a,9-dimethyl-3-methylidene-4,5,6,7,9a,9b-hexahydro-*3aH*-benzo[g][1]benzofuran-2-one	C_15_H_20_O_3_	248	4290-13-5	Sesquiterpene lactones
46	32.32	397,265,010	5.95	hexadecanoic acid	C_16_H_32_O_2_	256	57-10-3	Long-chain fatty acids
47	32.74	24,016,622	0.36	hexadecan-2-ol	C_16_H_34_O	242	14852-31-4	Long-chain fatty alcohols
48	34.505	68,419,854	1.03	(*E*,*7R*,*11R*)-3,7,11,15-tetramethylhexadec-2-en-1-ol	C_20_H_40_O	296	150-86-7	Diterpenoids
49	35.855	689,642,609	10.33	(*9Z*,*12Z*,*15Z*)-octadeca-9,12,15-trienoic acid	C_18_H_30_O_2_	278	463-40-1	Linolenic/polyunsaturated fatty acid and derivatives
50	36.062	92,496,493	1.38	Octadecanoic acid	C_18_H_36_O_2_	284	57-11-4	Long-chain fatty acids
51	37.686	38,938,196	0.58	2-methylhexadecan-1-ol	C_17_H_36_O	256	2490-48-4	Long-chain fatty alcohol
52	38.058	22,225,928	0.33	(4*aS*,10*aS*)-1,1,4*a*-trimethyl-3,4,10,10a-tetrahydro-*2H*-phenanthren-9-one	C_17_H_22_O	242	Not found	Diterpenoids
53	38.25	32,679,936	0.48	3-hydroxydodecanoic acid	C_12_H_24_O_3_	216	1883-13-2	Medium-chain hydroxy acids and derivatives
54	38.606	22,710,108	0.34	1-(3*a*,6,8*b*-trimethyl-1,2,3,4-tetrahydrocyclopenta[a]inden-7-yl)ethanone	C_17_H_22_O	242	Not found	Indene and cyclopentane-fused aromatic ketone derivatives
55	38.962	33,055,423	0.49	4-methoxy-6-[(*E*)-2-(3-methylphenyl)ethenyl]pyran-2-one	C_15_H_14_O_3_	242	Not found	Kavalactones
56	40.086	332,613,049	4.97	1,8-dihydroxyanthracene-9,10-dione	C_14_H_8_O_4_	240	117-10-2	Anthraquinone derivative
57	41.105	132,674,090	1.98	Octadecanedioic acid	C_18_H_34_O_4_	314	871-70-5	Long-chain fatty acids
58	41.296	20,610,941	0.31	2,3-dihydroxypropyl decanoate	C_13_H_26_O_4_	246	2277-23-8	Monoacylglycerol
59	41.982	74,761,965	1.12	1,3-dihydroxypropan-2-yl hexadecanoate	C_19_H_38_O_4_	330	23470-00-0	Monoacylglycerol
60	43.606	15,567,068	0.23	(*2E*,*6E*,*10E*)-3,7,11,15-tetramethylhexadeca-2,6,10,14-tetraen-1-ol	C_20_H_34_O	290	24034-73-9	Diterpenoid
61	45.904	182,945,069	2.74	[(*2S*)-2,3-dihydroxypropyl] (*9Z*,*12Z*,*15Z*)-octadeca-9,12,15-trienoate	C_21_H_36_O_4_	352	122-43-0	Linolenic/polyunsaturated fatty acid and derivatives
62	46.473	107,908,030	1.62	[(*2S*)-2,3-dihydroxypropyl] (*9Z*,*12Z*)-octadeca-9,12-dienoate	C_21_H_38_O_4_	354	3443-82-1	Linolenic/polyunsaturated fatty acid and derivatives
63	49.223	36,452,731	0.55	(*8Z*,*11Z*,*14Z*)-Icosa-8,11,14-trienoic acid	C_20_H_34_O_2_	306	1783-84-2	Polyunsaturated fatty acid
64	52.185	174,528,809	2.61	(*3S*,*8S*,*9S*,*10R*,*13R*,*14S*,*17R*)-17-[(*2R*,*5R*)-5-ethyl-6-methylheptan-2-yl]-10,13-dimethyl-2,3,4,7,8,9,11,12,14,15,16,17-dodecahydro-*1H*-cyclopenta[a]phenanthren-3-ol	C_29_H_50_O	414	83-46-5	Phytosterol
65	52.898	222,090,153	3.32	(*3S*,*4aR*,*6aR*,*6bS*,*8aR*,*12aR*,*14aR*,*14bR*)-4,4,6a,6b,8a,11,11,14b-octamethyl-1,2,3,4a,5,6,7,8,9,10,12,12a,14,14a-tetradecahydropicen-3-ol	C_30_H_50_O	426	559-70-6	Triterpenoids
66	55.622	44,176,754	0.66	(*4aS*,*6aR*,*6bS*,*8aR*,*12aR*,*14aR*,*14bS*)-4,6a,6b,8a,11,11,14b-Heptamethyl-2,4a,5,6,7,8,9,10,12,12a,14,14a-dodecahydro-*1H*-picene	C_29_H_46_	394	201358-24-9	Triterpenoids
67	56.813	72,458,639	1.08	1,2,4a,6a,6b,9,9,12a-Octamethyl-2,3,4,5,6,6a,7,8,8a,10,11,12,13,14b-tetradecahydro-*1H*-picene	C_30_H_50_	410	2933-32-6	Triterpene
68	57.558	89,616,481	1.34	(*1R*,*3aR*,*5aR*,*5bR*,*7aR*,*9S*,*11aR*,*11bR*,*13aR*,*13bR*)-3a,5a,5b,8,8,11a-Hexamethyl-1-(prop-1-en-2-yl)-1,2,3,4,5,6,7,7a,9,10,11,11b,12,13,13a,13b-hexadecahydrocyclopenta[a]chrysen-9-ol	C_30_H_50_O	426	545-47-1	Triterpenoid

**Table 3:** ZoI (mm), MIC, and MBC values of FALE.

Bacterium/ Dilution	Chloramphenicol, 25 µg/mL	1000 μg/mL	500 μg/mL	250 μg/mL	125 μg/mL	MIC (μg/mL)	MBC (μg/mL)
*S. aureus*	22 ± 1	20 ± 2 *	18 ± 2 *	15 ± 2 *	12 ± 2 *	15.63 ± 1.76	31.25 ± 1.82
*E. faecalis*	23 ± 1	21 ± 1 *	18 ± 2 *	15 ± 1 *	13 ± 1 *	31.25 ± 1.82	62.50 ± 1.55
*Bacillus subtilis*	20 ± 2	18 ± 2 *	15 ± 1 *	13 ± 1 *	10 ± 2 *	31.25 ± 1.82	62.50 ± 1.55
*E. coli*	20 ± 1	15 ± 2 *	13 ± 1 *	10 ± 1 *	8 ± 2 *	62.50 ± 0.76	125 ± 1.37
*K. pneumoniae*	19 ± 2	16 ± 2 *	14 ± 1 *	11 ± 1 *	9 ± 2 *	62.50 ± 0.76	125 ± 1.37
*P. aeruginosa*	21 ± 1	17 ± 1 *	15 ± 1 *	14 ± 1 *	9 ± 0 *	62.50 ± 0.76	125 ± 1.37

Note: Zones of inhibition (ZoI), minimum inhibitory concentration (MIC), minimum bactericidal concentration (MBC)**.** Values are presented as mean ± SD of triplicate measurements. The results demonstrate a statistically significant decrease relative to the positive control (25 µg/mL chloramphenicol), as indicated by (* = *p* < 0.05).

**Table 4:** The ZoI (mm), MIC (μg/mL), and MBC (μg/mL) of FASE.

Bacterium/ Dilution	Chloramphenicol, 25 µg/mL	1000 μg/mL	500 μg/mL	250 μg/mL	125 μg/mL	MIC (μg/mL)	MBC (μg/mL)
*S. aureus*	22 ± 1	21 ± 1 *	19 ± 0 *	16 ± 2 *	14 ± 2 *	7.81 ± 0.36	15.63 ± 1.22
*E. faecalis*	23 ± 1	22 ± 2 *	20 ± 2 *	17 ± 1 *	15 ± 1 *	15.63 ± 1.22	31.25 ± 0.34
*B. subtilis*	20 ± 2	20 ± 2 *	17 ± 1 *	15 ± 1 *	13 ± 1 *	15.63 ± 0.78	31.25 ± 0.78
*E. coli*	20 ± 1	17 ± 1 *	15 ± 0 *	13 ± 1 *	10 ± 1 *	15.63 ± 1.77	31.25 ± 1.18
*K. pneumoniae*	19 ± 2	17 ± 1 *	15 ± 1 *	11 ± 2 *	10 ± 0 *	31.25 ± 1.27	62.50 ± 1.35
*P. aeruginosa*	21 ± 1	19 ± 1 *	16 ± 1 *	15 ± 1 *	11 ± 2 *	31.25 ± 1.36	62.50 ± 1.37

Note: Zones of inhibition (ZoI), minimum inhibitory concentration (MIC), minimum bactericidal concentration (MBC). Values are presented as mean ± SD of triplicate measurements. The results demonstrate a statistically significant decrease from the positive control (chloramphenicol 25 µg/mL), indicated by (* = *p* < 0.05).

**Table 5:** The ZoI, MIC, and minimum fungicidal concentration (MFC) values of FALE against selected *Candida* strains.

Fungal/ Dilution	Fluconazole (25 µg/mL)	1000 μg/mL	500 μg/mL	250 μg/mL	125 μg/mL	MIC (μg/mL)	MFC (μg/mL)
*Candida albicans*	23 ± 1	19 ± 2 *	16 ± 1 *	14 ± 1 *	12 ± 2 *	31.25 ± 0.33	62.50 ± 0.45
*C. glabrata*	22 ± 1	18 ± 1 *	16 ± 1 *	14 ± 1 *	12 ± 1 *	31.25 ± 2.61	62.50 ± 1.63
*C. parapsilosis*	21 ± 2	19 ± 1 *	17 ± 1 *	15 ± 0 *	13 ± 2 *	31.25 ± 0.78	62.50 ± 1.28

Note: Zones of inhibition (ZoI), minimum inhibitory concentration (MIC), minimum fungicidal concentration (MFC). The reported values are shown in triplicate as mean ± SD. The results demonstrate a statistically significant decrease from the positive control (25 µg/mL fluconazole, as indicated by (* = *p* < 0.05)).

**Table 6:** The ZoI, MIC, and MFC values of FASE against selected *Candida* strains.

Fungal/ Dilution	Fluconazole (25 µg/mL)	1000 μg/mL	500 μg/mL	250 μg/mL	125 μg/mL	MIC (μg/mL)	MFC (μg/mL)
*Candida albicans*	23 ± 1	21 ± 1 *	18 ± 2 *	16 ± 1 *	13 ± 1 *	15.63 ± 1–52	31.25 ± 1.38
*C. glabrata*	22 ± 1	19 ± 2 *	17 ± 2 *	15 ± 1 *	13 ± 1 *	15.63 ± 1–67	31.25 ± 1.48
*C. parapsilosis*	21 ± 2	20 ± 2 *	18 ± 2 *	16 ± 1 *	14 ± 1 *	15.63 ± 1–57	31.25 ± 1.13

Note: Zones of inhibition (ZoI), minimum inhibitory concentration (MIC), minimum fungicidal concentration (MFC). The reported values are shown in triplicate as mean ± SD. The results demonstrate a statistically significant decrease from the positive control (25 µg/mL fluconazole, as indicated by (* = *p* < 0.05)).

## Data Availability

The original contributions presented in this study are included in the article. Further inquiries can be directed to the corresponding author.

## Abbreviations

The following abbreviations are used in this manuscript:

GC–MS	Gas Chromatography–Mass Spectrometry
TPC	Total Phenolic Content
TFC	Total Flavonoid Content
DPPH	2,2-Diphenyl-1-picrylhydrazyl
ABTS	2,2’-Azino-*bis*(3-ethylbenzothiazoline-6-sulfonic acid)
MIC	Minimum Inhibitory Concentration
MBC	Minimum Bactericidal Concentration
